# Dissection of the TssB-TssC Interface during Type VI Secretion Sheath Complex Formation

**DOI:** 10.1371/journal.pone.0081074

**Published:** 2013-11-25

**Authors:** Xiang Y. Zhang, Yannick R. Brunet, Laureen Logger, Badreddine Douzi, Christian Cambillau, Laure Journet, Eric Cascales

**Affiliations:** 1 Laboratoire d′Ingénierie des Systèmes Macromoléculaires (LISM, UMR 7255), Institut de Microbiologie de la Méditerranée (IMM), Centre National de la Recherche Scientifique (CNRS), Aix-Marseille Université, Marseille, France; 2 Architecture et Fonction des Macromolécules Biologiques (AFMB, UMR 6098), Centre National de la Recherche Scientifique (CNRS), Aix-Marseille Université, Marseille, France; The University of Texas at San Antonio, United States of America

## Abstract

The Type VI secretion system (T6SS) is a versatile machine that delivers toxins into either eukaryotic or bacterial cells. At a molecular level, the T6SS is composed of a membrane complex that anchors a long cytoplasmic tubular structure to the cell envelope. This structure is thought to resemble the tail of contractile bacteriophages. It is composed of the Hcp protein that assembles into hexameric rings stacked onto each other to form a tube similar to the phage tail tube. This tube is proposed to be wrapped by a structure called the sheath, composed of two proteins, TssB and TssC. It has been shown using fluorescence microscopy that the TssB and TssC proteins assemble into a tubular structure that cycles between long and short conformations suggesting that, similarly to the bacteriophage sheath, the T6SS sheath undergoes elongation and contraction events. The TssB and TssC proteins have been shown to interact and a specific α-helix of TssB is required for this interaction. Here, we confirm that the TssB and TssC proteins interact in enteroaggregative *E. coli*. We further show that this interaction requires the N-terminal region of TssC and the conserved α-helix of TssB. Using site-directed mutagenesis coupled to phenotypic analyses, we demonstrate that an hydrophobic motif located in the N-terminal region of this helix is required for interaction with TssC, sheath assembly and T6SS function.

## Introduction

The Type VI secretion system (T6SS) is a widely distributed secretion system in proteobacteria [1]–[3]. Initially identified as a system allowing *Vibrio cholerae* to resist predation by amoeba, recent data have demonstrated that it allows virulence towards eukaryotic host cells and/or competition towards neighbouring bacteria [4]–[13]. Among the effector domains delivered into eukaryotic host cells, the best characterized is the C-terminal domain of the VgrG protein of *V. cholerae*, that cross-links G-actin hence abrogating cytoskeleton rearrangements and dynamics [14]–[16]. T6SS effectors delivered into bacterial cells have been shown to bear peptidoglycan hydrolysis or phospholipase activities [6], [17]–[24]. At a molecular level, the T6SS is composed of a membrane complex comprising four essential components, the TssL and TssM inner membrane proteins, the TssK cytoplasmic protein and the TssJ outer membrane lipoprotein [25]–[33]. This membrane complex serves as anchor for a cytoplasmic tubular structure that is structurally and functionally similar to the tails of contractile bacteriophages [34]–[37]. This tubular structure is composed of at least 5 components that share similarities with bacteriophage subunits. The tails of contractile bacteriophages are composed of an internal tube that polymerizes onto a baseplate composed of six wedges and of the hub complex – or cell-puncturing device [38], [39]. This internal tube is wrapped by a sheath structure that contracts upon attachment allowing to propel the internal tube towards the host cell [39]. The Hcp protein shares its fold with that of gpV, the tail tube protein of bacteriophage λ [40], [41] whereas the VgrG protein assemble into a homotrimeric structure resembling the trimeric gp27-gp5 hub complex of bacteriophage T4 [14], [35], [42], [43]. TssE is structurally similar to gp25, a component of the baseplate wedges [1], [44]. Finally, TssB and TssC assemble into tubular structures with cogwheel patterns resembling the bacteriophage contractile sheath [45], [46]. Indeed, cryo-electron tomography analyses showed that this structure exists either in an extended and thin conformation or in a short but wider conformation [47], [48]. Fluorescence microscopy experiments further showed that these tubular structures are dynamic and oscillate between long and short conformations before being disassembled [13], [47]–[49]. This mechanism is reminiscent of the bacteriophage sheath, which undergoes a contraction event when the viral particle attaches on host cell. Based on the homologies between the bacteriophage tail and several T6SS components, it is thought that the cytoplasmic tubular structure is composed of an internal tube formed of hexamers of the Hcp protein stacked onto each other and wrapped by the TssB and TssC subunits that assemble into a sheath. Upon contact with a target cell, the sheath will contract, propelling the Hcp tube terminated by the VgrG protein towards the target cell [34]–[36]. It has been recently shown that contraction of the T6SS sheath coincides with the lysis of the target bacterial cell [13]. Finally, sheath disassembly is catalysed by ClpV, a specialized ATPase associated with the T6SS [45], [47]–[50]. ClpV interacts with an α-helix of TssC after contraction and is thought to either recycle the TssB and TssC proteins for a new round of assembly or target both proteins to degradation [48], [51]. The interaction between the TssB and TssC subunits has been demonstrated in several microorganisms including *Francisella tularensis*, *Vibrio cholerae*, *Burkholderia cenocepacia*, *Yersinia pseudotuberculosis*, *Salmonella enterica* Typhimurium, and *Pseudomonas aeruginosa* [45], [46], [52]–[54]. The N-terminal 212 amino-acids of TssC are required for the TssB-TssC interaction in *B. cenocepacia* and in *P. aeruginosa* [46], [54] while an α-helix located between residues 100–130 of TssB are necessary in *F. tularensis*, *V. cholerae* and *P. aeruginosa* [52], [55]. Here, we confirm the interaction between the TssB and TssC proteins encoded by the *sci-1* T6SS gene cluster of enteroaggregative *Escherichia coli* (EAEC) and that the N-terminal region of TssC and the α-helix of TssB are required for the interaction. Finally, we dissect the role of α-helix residues and demonstrate that an hydrophobic side of the helix located in its N-terminal region is necessary for the TssB-TssC interaction, sheath assembly and T6SS function.

## Results

### TssB interacts with the N-terminal domain of TssC

**Figure 1:**
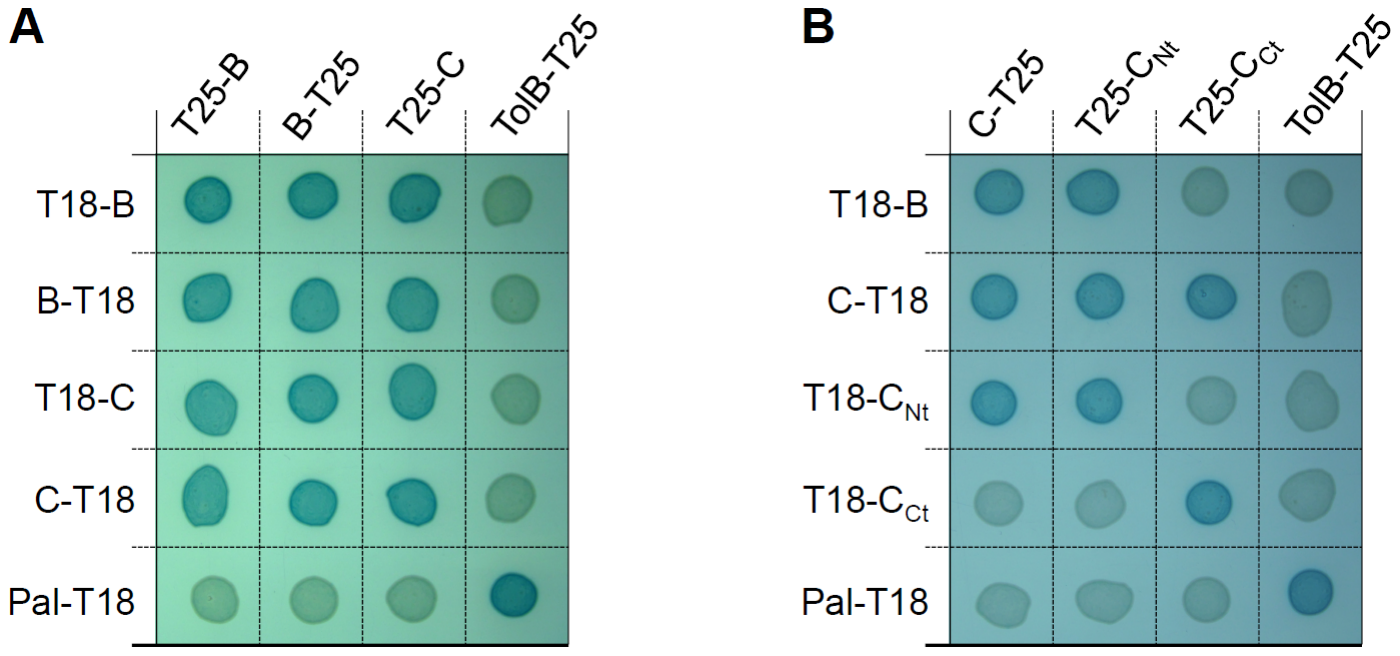
TssB1 interacts with the N-terminal domain of TssC1. BTH101 reporter cells carrying pairs of plasmids producing the indicated T6SS proteins or domains (B, TssB1; C, TssC1; C_Nt_, N-terminal domain of TssC1; C_Ct_, C-terminal domain of TssC1) fused to the T18 or T25 domain of the *Bordetella* adenylate cyclase were spotted on X-Gal indicator LB agar plates. Controls include T18 and T25 fusions to TolB and Pal, two proteins that interact but unrelated to the T6SS.

The TssB and TssC proteins assemble into a sheath structure that has been proposed to wrap an internal tube constituted of stacked Hcp hexamers. The interaction between these two proteins has been demonstrated in a number of bacterial strains including *V. cholerae*, *S. enterica* Typhimurium, *Y. pseudotuberculosis*, *F. tularensis*, *P. aeruginosa* and *B. cenocepacia* [44], [45], [51]–[53]. In agreement with these previous studies, we showed that TssB1 (accession numbers: EC042_4524; YP_006098801) and TssC1 (accession numbers: EC042_4525; YP_006098802), two proteins encoded within the *sci-1* T6SS gene cluster in enteroaggregative *E. coli* interact in a bacterial two-hybrid assay (Fig. 1A). We also observed that TssB1 and TssC1 oligomerize, as TssB1/TssB1 and TssC1/TssC1 combinations activated the expression of the reporter gene (Fig. 1A). Regarding TssC proteins, it has been shown in *P. aeruginosa* and *B. cenocepacia* that the N-terminal fragment comprising the first 212 amino-acids were sufficient for the interaction with TssB [46], [54]. HHPred and Phyre domain analyses of TssC1 suggested that it is composed of two domains, including an N-terminal region of 384 amino-acids (residues 1–384) and a C-terminal region of 130 amino-acids (residues 385–514). We generated constructions to test the interaction between TssB1 and the N-terminal (1-384) or C-terminal (385–514) domains of TssC1. As shown in Fig. 1B, the N-terminal region of TssC1 was sufficient to interact with TssB1. Interestingly, we also noted that both the N- and C-terminal domains oligomerizes. However, no interaction between the N-terminal and C-terminal domains of TssC1 was detected (Fig. 1B).

### An α-helix of TssB is required for T6SS function, TssC interaction but not for self-interaction

**Figure 2:**
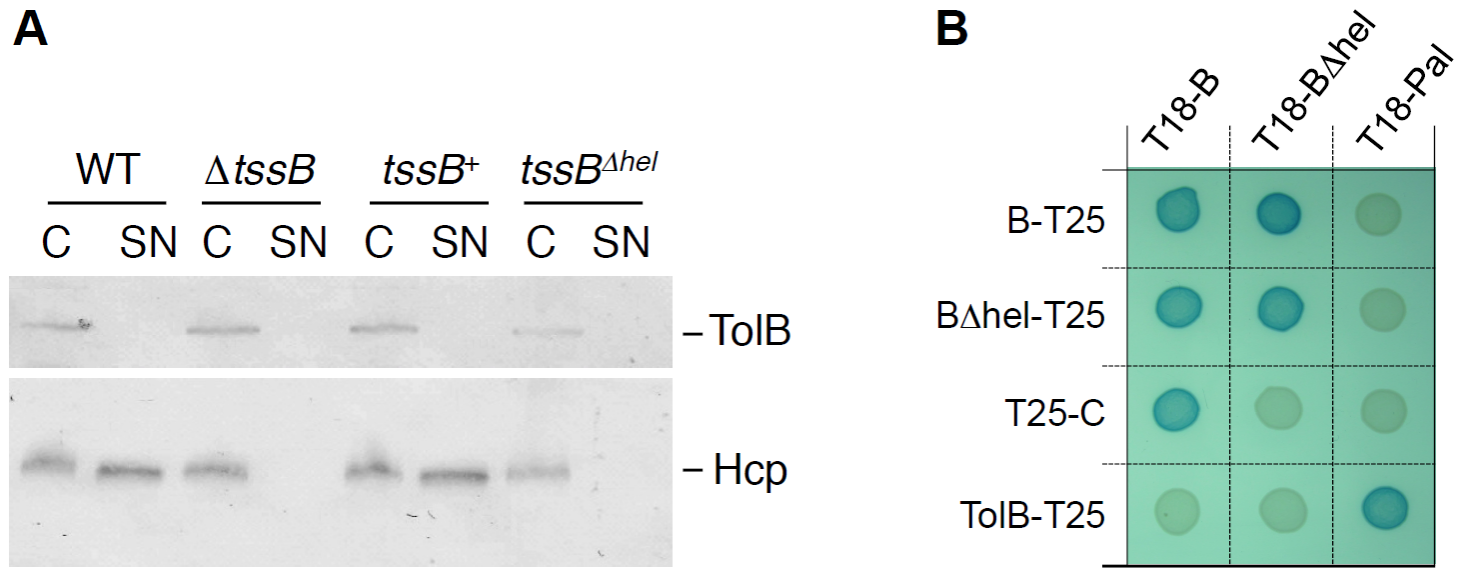
The α-helix located between residues 104 and 130 of TssB1 is required for T6SS function and interaction with TssC1. (A) Hcp release assay. Hcp_FLAG_ release was assessed by separating whole cells (C) and culture supernatant (SN) fractions from 2×10^9^ wild-type (WT), Δ*tssB1* (Δ*tssB*) cells or Δ*tssB1* cells producing TssB1 (*tssB^+^*) or TssB1 deleted of the α-helix 104-130 (*tssB*Δ*^hel^*). Proteins were separated by 12.5%-acrylamide SDS-PAGE and Hcp was immunodetected using anti-FLAG monoclonal antibody (lower panel). The periplasmic TolB protein (immunodetected using an anti-TolB polyclonal antibodies, upper panel) was used as a marker to verify that no lysis occured. (B) Bacterial two-hybrid assay. BTH101 reporter cells carrying pairs of plasmids producing the indicated T6SS proteins (B, TssB1; BΔhel, TssB1 deleted of the α-helix; C1, TssC1) fused to the T18 or T25 domain of the *Bordetella* adenylate cyclase were spotted on X-Gal indicator LB agar plates.

In *Francisella*, an α-helix of the TssB homologue IglA, located between residues 103 and 122, has been shown to be essential for the function of the FPI-encoded T6SS [52]. Yeast two-hybrid experiments further demonstrated that this region is required for efficient interaction with IglB, the *F. tularensis* homologue of TssC [52]. The mode of recognition between IglA and IglB has been shown to be conserved as the interactions between the *P. aeruginosa* HSI-3, *Y. pseudotuberculosis*, *V. cholerae* and uropathogenic *E. coli* TssB and TssC proteins require this α-helix [52], [55]. Our results show that this is indeed conserved in EAEC, as deletion of the helix residues located between residues E104 and L131 of TssB1 abolishes the ability of the Sci-1 T6SS to release the Hcp1 protein (Fig. 2A) and to interact with TssC1 (Fig. 2B). However, bacterial two-hybrid experiments showed that deletion of this α-helix has no impact on TssB oligomerization (Fig. 2B).

### The hydrophobic face of the α-helix is required for TssC interaction and assembly of the sheath-like structure

**Figure 3:**
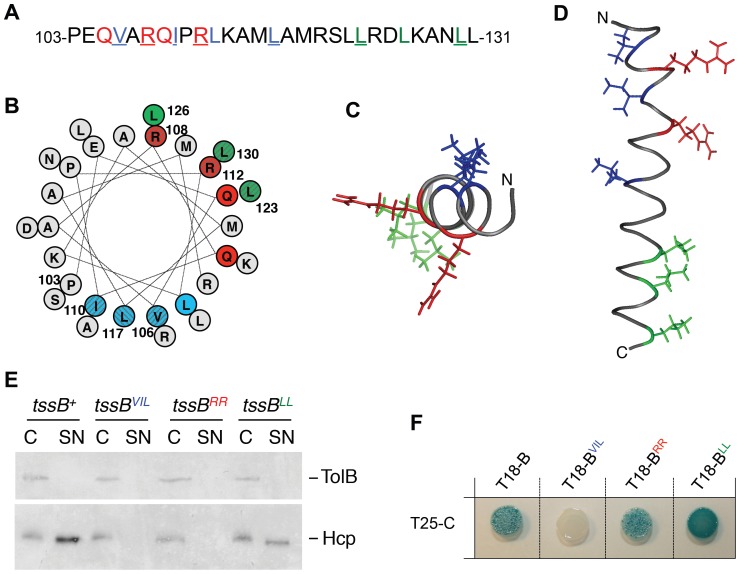
The hydrophobic motif of the TssB1 α-helix is required for T6SS function and interaction with TssC1. Amino-acid sequence (A) and helical wheel projection (B) of the TssB1 α-helix (from residue Pro-103 to residue Leu-131). The different motifs described in this study are highlighted in different colours: blue, N-terminal hydrophobic; red, polar/charged; green, leucine-rich. The residues of these motifs mutagenized in this study are underlined (A) or striped (B). Top-view (C) and side-view (D) projections of the TssB1 α-helix. The targeted residues are colored as in panel (A). Top- and side-views have been modelled using PyMOL v0.99. (E) Hcp release assay. Hcp_FLAG_ release was assessed by separating whole cells (C) and culture supernatant (SN) fractions from 2×10^9^ Δ*tssB1* cells producing TssB1 (*tssB^+^*) or TssB1 bearing substitutions within the hydrophobic (*tssB^VIL^*), the polar/charged (*tssB^RR^*) or the leucin-rich (*tssB^LL^*) motif. Proteins were separated by 12.5%-acrylamide SDS-PAGE and Hcp and TolB were immunodetected using anti-FLAG monoclonal (lower panel) and anti-TolB polyclonal (upper panel) antibodies. (F) Bacterial two-hybrid assay. BTH101 reporter cells producing the T25 domain of the *Bordetella* adenylate cyclase fused to TssC1 (T25-C) and the T18 domain fused to TssB1 (T18-B) or TssB1 variants bearing substitutions within the hydrophobic (T18-B^VIL^), the polar/charged (T18-B^RR^) or the leucin-rich (T18-B^LL^) motif were spotted on X-Gal indicator LB agar plates.

**Figure 4:**
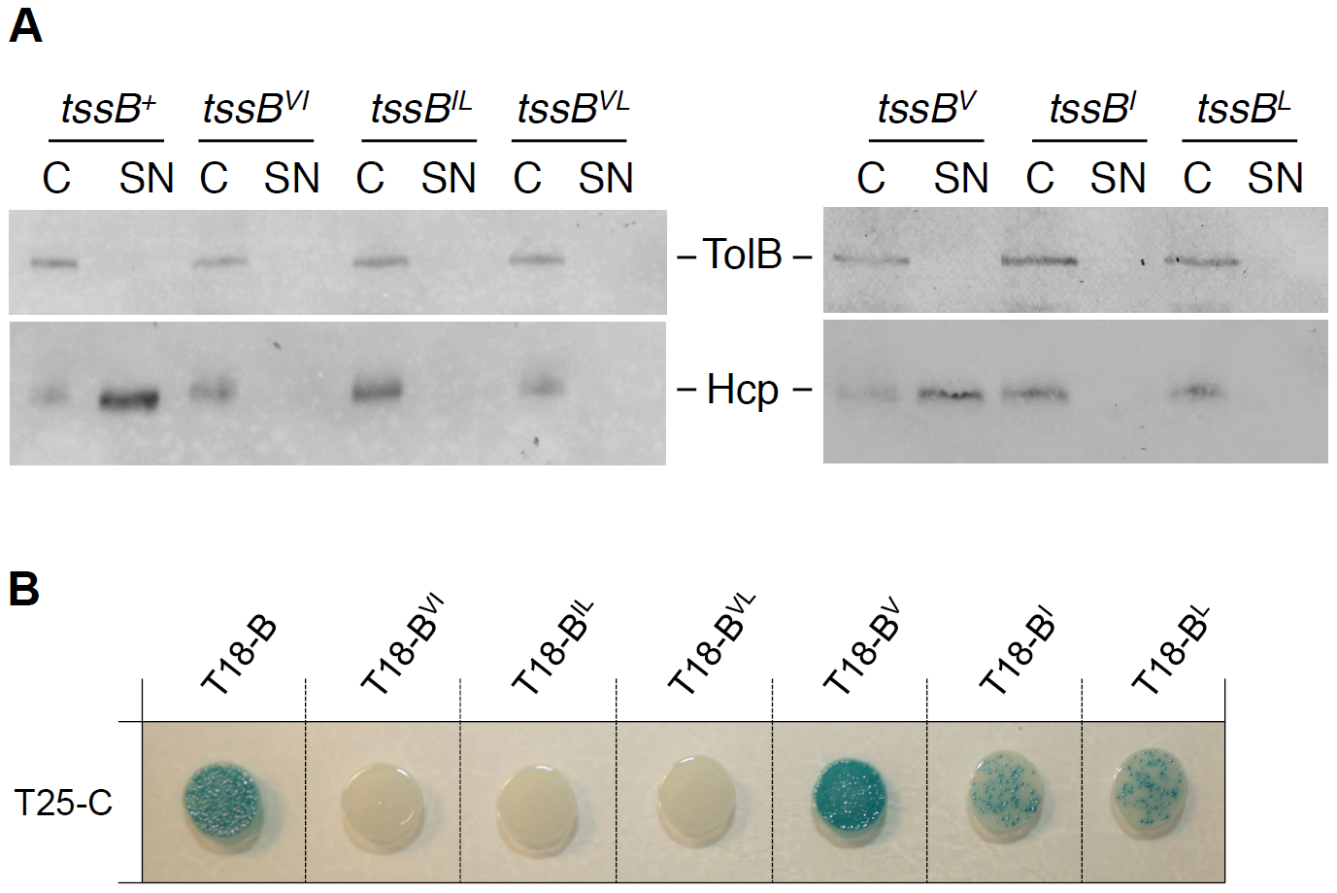
Mutagenesis study of the hydrophobic motif of the TssB1 α-helix. (A) Hcp release assay. Hcp_FLAG_ release was assessed by separating whole cells (C) and culture supernatant (SN) fractions from 2×10^9^ Δ*tssB1* cells producing TssB1 (*tssB^+^*) or TssB1 bearing double or single substitutions within the hydrophobic motif (*tssB^VI^*, V106W-I110W; *tssB^IL^*, I110W-L117W; *tssB^VL^*, V106W-L117W; *tssB^V^*, V106W; *tssB^I^*, I110W; *tssB^L^*, L117W). Proteins were separated by 12.5%-acrylamide SDS-PAGE and Hcp and TolB were immunodetected using anti-FLAG monoclonal (lower panel) and anti-TolB polyclonal (upper panel) antibodies. (B) Bacterial two-hybrid assay. BTH101 reporter cells producing the T25 domain of the *Bordetella* adenylate cyclase fused to TssC1 (T25-C) and the T18 domain fused to TssB1 (T18-B) or TssB1 variants bearing substitutions within the hydrophobic motif (T18-B^VI^, V106W-I110W; T18-B^IL^, I110W-L117W; T18-B^VL^, V106W-L117W; T18-B^V^, V106W; T18-B^I^, I110W; T18-B^L^, L117W) were spotted on X-Gal indicator LB agar plates.

The sequence of the α-helix of the EAEC TssB1 protein facilitating its interaction with TssC1 is shown in Fig. 3A. Interestingly, a projection of this α-helix (Fig. 3B, 3C and 3D) showed that the N-terminal region presents an amphipathic character as it comprises both a hydrophobic side (Val-106, Ile-110, Leu-113 and Leu-117) and a polar/charged side (Glu-104, Gln-105, Arg-108, Gln-109, Arg-112, Arg-120). Finally, one face of the C-terminal region of the helix is rich in leucine residues (Leu-122, Leu-123, Leu-126, Leu-130) resembling a leucine-zipper motif (Fig. 3B, 3C and 3D). To better understand the mode of binding between the TssB and TssC proteins, we engineered TssB1 variants in which the hydrophobic, polar/charged and leucine motifs were substituted by tryptophan residues (the residues targeted in this study are highlighted in colour in Fig. 3A-D). Tryptophan-scanning mutagenesis is usually used to test interactions between α-helices as tryptophan residues do not disrupt formation of α-helices but induces a steric hindrance abolishing protein-protein contacts [56], [57]. Mutations within the leucine-rich motif (L123W-L130W, called LL) had no effect on Sci-1 T6SS function and on TssB1-TssC1 interaction as shown by Hcp release (Fig. 3E) and two-hybrid (Fig. 3F) assays. Interestingly, while substitution of the polar/charged motif (R108W-R112W, called RR) had no effect on TssB1-TssC1 interaction, the function of the T6SS was abrogated, suggesting that these mutations may affect interactions with other partners or proper folding of TssB1. Finally, mutations within the hydrophobic motif (V106W-I110W-L117W) abolished both the release of Hcp and the interaction with TssC1 (Fig. 3E and 3F). We further dissected the effect of the hydrophobic motifs by generating double and single substitutions (Fig. 4). All the double substitutions within the hydrophobic faceabrogated T6SS function and TssB1-TssC1 interaction. Regarding the single substitutions, mutation of Val-106 had no effect while mutations of Ile-110 and Leu-117 abolished T6SS function (Fig. 4A) by decreasing TssB1 binding to TssC1 (Fig. 4B). It is worth noting that all the TssB1 variants used in this study accumulated at levels comparable to the wild-type TssB1 protein (data not shown).

**Figure 5:**
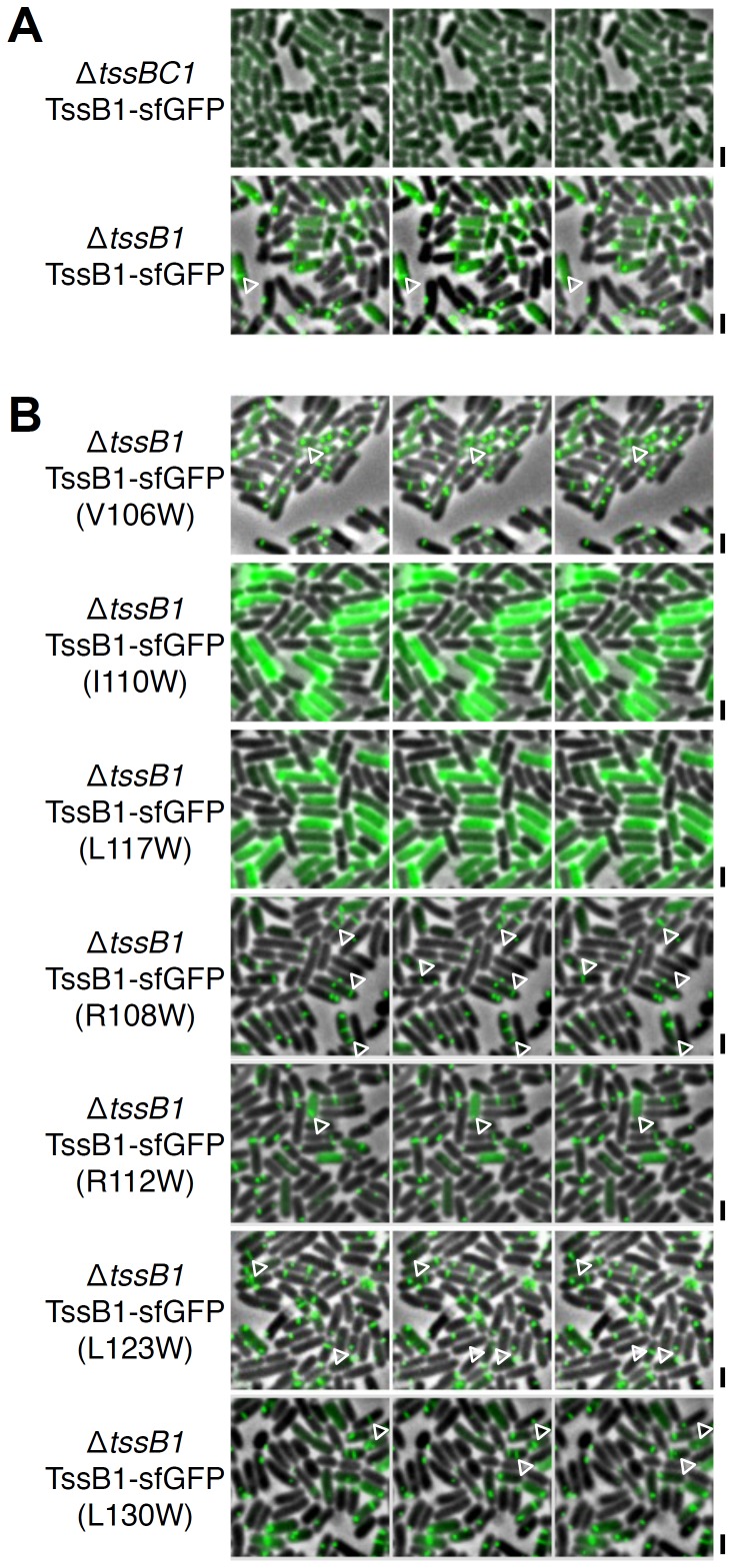
The hydrophobic motif of the TssB1 α-helix is required for T6SS sheath assembly. Time-lapse fluorescence microscopy recordings showing sheath dynamics in Δ*tssBC1* (Δ*tssB1*-Δ*tssC1*) or Δ*tssB1* cells producing TssB1-sfGFP (TssB1) (A) or TssB1-sfGFP bearing the indicated substitutions (B). Individual images were taken every 30 sec. Assembly and contraction events are indicated by the white open triangles. Scale bars are 2 µm.

Finally, we wondered whether disruption of the TssB-TssC interaction impacts sheath assembly. Fluorescence microscopy approaches have recently been developed to follow the assembly of TssBC sheath structures by fusing TssB to the superfolder Green Fluorescent Protein (sf-GFP) [13], [33], [47]–[50]. In Δ*tssB1* cells producing TssB1-sfGFP, the fusion protein assembles into tubular structures that undergo cycles of assembly, contraction and disassembly (Fig. 5A). These structures are not visible in Δ*tssBC1* cells (Fig. 5A), in agreement with the fact that these tubular structures are composed of both TssB and TssC proteins [45], [46]. However, when the overall fluorescence levels of Δ*tssB1* or Δ*tssBC1* cells producing TssB1-sfGFP were measured, we observed that Δ*tssBC1* cells displayed 6–8% of fluorescence compared to Δ*tssB1* cells, suggesting that TssB1-sfGFP is less stable in absence of TssC1. When TssB1-sfGFP variants bearing single substitutions within the polar/charged or leucine-rich motif were produced in Δ*tssB1* cells, dynamic structures oscillating between extended and contracted conformations were observed, indicating a functional T6SS (Fig. 5B). By contrast, mutations of the Ile-110 or Leu-117 residue led to a diffuse fluorescence in the cell body without any identifiable tubular structures, demonstrating that disruption of the TssB-TssC interaction prevents formation of T6SS sheaths (Fig. 5B).

## Discussion

The portion of the T6SS related to the bacteriophage tail is composed of an internal tube comprising hexameric rings of Hcp stacked onto each other and wrapped into a structure formed by the TssB and TssC components. The assembly of this structure oscillates between elongated and contracted conformations and is thought to be similar to the mechanism of action of bacteriophage contractile sheaths. It has been therefore proposed that conformational changes in the TssB and TssC proteins induce a large modification in this structure leading to its contraction and allowing propelling of the internal tube towards the exterior [34], [35], [43], [45], [46]. The internal tube is tipped by the VgrG protein that acts as a cell-puncturing device to perforate the host cell membrane allowing delivery of effector domains or proteins [34], [36], [37]. In this work, we focussed our attention on the TssB1 and TssC1 proteins encoded within the *sci-1* gene cluster of enteroaggregative *E. coli*.

Using bacterial two-hybrid experiments, we showed that (i) TssB1 interacts with itself, (ii) TssC1 interacts with itself, and (iii) TssB1 and TssC1 interact. The self-interaction of the TssB and TssC components is in agreement with the formation of long structures that should comprise hundreds of TssB and TssC molecules. We also showed that the N-terminal region of TssC1 and the C-terminal region of TssC1 self-interact. However, we did not detect interaction between the N-terminal and C-terminal regions of TssC1, suggesting that TssC1 oligomeric complex formation is driven by N-terminal/N-terminal and C-terminal/C-terminal interactions. We also showed that the N-terminal domain of TssC1 is sufficient for its interaction with TssB1. The TssB-TssC interaction was previously demonstrated in numerous studies [45], [46], [52]–[54] and the requirement of the N-terminal domain of TssC for the interaction was evidenced in *B. cenocepacia* and *P. aeruginosa* [46], [54]. The interaction between TssB and TssC has been shown to stabilize both proteins [45], [46], [52], [55]. Interestingly, our fluorescence microscopy experiments showed that the level of fluorescence of Δt*ssB1C1* cells producing TssB1-sfGFP is 6–8% of the fluorescence in Δ*tssB1* cells, confirming that TssB-sfGFP is stabilized by the presence of TssC.

The TssB1 protein of EAEC is composed of 165 amino-acids. Secondary structure predictions showed that it contains an α-helix between residues Pro-103 and Leu-131. This α-helix has been previously shown to be essential for T6SS function and TssB-TssC interaction in *F. tularensis* and *V. cholerae* [52], [55]. Interestingly, the authors of this study also showed that this α-helix is essential for TssB-TssC interaction in several microorganisms including *V. cholerae*, *Y. pseudotuberculosis*, *S. enterica* Typhimurium, uropathogenic *E. coli* and *P. aeruginosa*, suggesting that the mode of binding of TssB to TssC is conserved [52]. We obtained similar results in enteroaggregative *E. coli*: deletion of this conserved α-helix causes disruption of the TssB1-TssC1 interaction and abolishes T6SS function as shown by Hcp release assay. When projected on a helical wheel, we observed that this α-helix has a strong amphipathic character in its N-terminal region. Indeed, one face of the helix is essentially constituted of hydrophobic residues (Val, Leu, Ile) while the opposite face is composed of polar or positively charged residues (Arg, Gln). The charge partition in the C-terminal region of the helix is better balanced but we observed a leucine-rich face, on the same side that the polar/charged motif. Using site-directed mutagenesis, we substituted residues within each of these motifs. Our analyses highlighted the importance of the N-terminal hydrophobic motif. Mutation of the three Val-106, Ile-110 and Leu-117 residues showed that this motif is required for TssB1-TssC1 interaction, sheath assembly and T6SS function. Single and double substitutions further showed that the Ile-110 and Leu-117 residues have a more important contribution within this motif. Our results also indicated that substitution of the arginine-rich motif within the conserved α-helix does not prevent formation of the TssB1-TssC1 complex (Fig. 3F); however, the T6SS is not functional as shown by Hcp release assay (Fig. 3E). This suggests that mutation of this motif may disrupt interaction of TssB1 with additional partners, yet to be identified, or that it prevents proper folding of TssB1. Indeed, fluorescence microscopy experiments showed that mutation of these two arginine residues abolishes sheath assembly (data not shown). However, single substitutions had no effect on sheath assembly and on T6SS function. Interestingly, while preparing this manuscript, Bröms et al. published a study where they showed that a hydrophobic motif located in the same region of the *V. cholerae* TssB α-helix is required for proper interaction with TssC [55]. The results from this study and from the work described here showed that identical TssB motifs are required to functionally interact with TssC in *V. cholerae* and EAEC. We therefore hypothesize that the mode of binding of TssB to TssC is similar in T6SSs. However, Lossi et al. noted that the TssB and TssC proteins encoded by the *P. aeruginosa* HSI-1 T6SS gene cluster interact but are not able to interact with homologues encoded by a second T6SS gene cluster present on the chromosome [46]. One can hypothesize that the hydrophobic motif of the α-helix is the primary determinant for formation of the TssB-TssC complex but that additional determinants might control the proper and specific assembly of the sheath when several T6SS are produced at the same time. Further studies are required to better understand how TssB proteins identify their cognate partners.

## Experimental Procedures

### Bacterial strains, media and chemicals

*Escherichia coli* DH5α (New England Biolabs) was used for cloning procedures, BTH101 [58] for bacterial two-hybrid assays. Hcp release and fluorescence microscopy have been performed into the enteroaggregative *E. coli* strain 17-2. With the exception of the bacterial two-hybrid assay (at 30°C), cells were grown in Luria broth (LB) at 37°C. Plasmids were maintained by addition of ampicillin (100 µg/mL), chloramphenicol (40 µg/mL) or kanamycin (50 µg/mL). Gene expression was induced by 0.5 mM isopropyl-β-thio-galactoside (IPTG) or 0.01% arabinose. For the Hcp release assay, expression of the *sci-1* gene cluster was induced by addition of the iron chelator 2,2′-dipyridyl (125 µM final concentration) 30 minutes prior harvesting the cells [59]. For fluorescence microscopy experiments, cells were grown in minimal M9 medium supplemented with casaminoacids, glucose 0.4% and 10% of LB.

### Strain construction

Construction of the Δ*tssB1* and Δ*tssBC1* mutant strains. The *tssB1* gene was deleted into the enteroaggregative *E. coli* 17-2 strain using a modified one-step inactivation procedure [60] as previously described [25] using plasmid pKOBEG [61]. PCR products to be elctroporated were obtained using oligonucleotides DEL-4524-5/DW (5′-*GGAGGCATCTGCGGTGATGGAACCCCTGAGATGCAGGTTTCACAGGAGAGAGCC*TGTGTAGGCTGGAGCTGCTTCG) and DEL-4524-3/DW (5′-*TTCTTTTCTTTCTGTACAGACATCAGCATTTTCTCTCGTAATCCGTTAAA*CATATGAATATCCTCCTTAGTTCC) carrying 50- nucleotide extensions homologous to regions adjacent to *tssB1* (in italics). Kanamycin resistant clones were selected and verified by colony-PCR. The kanamycin cassette was then excised using plasmid pCP20 [60]. The deletion of *tssB1* was confirmed by colony-PCR. Construction of the Δ*tssBC1* strain was done identically, using oligonucleotides DEL-4524-5/DW and DEL-4525-3/DW (5′-*GCCCCGTCTTCCCATAATGGGCGATAAATCTTCATTTCCCGACACCTGCC*CATATGAATATCCTCCTTAGTTCC).

### Plasmid construction

Polymerase Chain Reactions (PCR) were performed using a Biometra thermocycler using Pfu Turbo DNA polymerase (Agilent Technology). Restriction enzymes were purchased from NewEngland Biolabs and used according to the manufacturer's instructions. Custom oligonucleotides were synthesized by Sigma Aldrich. Enteroaggregative *E. coli* 17-2 chromosomal DNA was used as a template for all PCRs. *E. coli* strain DH5α was used for cloning procedures. All the plasmids have been constructed by restriction-free cloning [62] as previously described [27]. Briefly, genes of interest were amplified with oligonucleotides introducing extensions annealing to the target vector. The double-stranded product of the first PCR has then been used as oligonucleotides for a second PCR using the target vector as template. Constructions of the bacterial two-hybrid vectors encoding TssB1 and TssC1 fusion proteins and of the pBAD33-TssB1-VSVG and pBAD-TssB1-sfGFP plasmids have been previously described [33]. Bacterial two-hybrid vectors encoding T18 and T25-TssC_Nt_ fusions were constructed by insertion of a premature stop codon into T18-TssC and T25-TssC by site directed mutagenesis using oligonucleotides 5′- GAACCGGGATTATGCCTGCTTCTTCTCTTAAGCTAACTCCGCCCAGAAACCGGCG and CGCCGGTTTCTGGGCGGAGTTAGCTTAAGAGAAGAAGCAGGCATAATCCCGGTTC (stop codon underlined). Bacterial two-hybrid vectors encoding T18- and T25-TssC_Ct_ fusions were obtained by restriction-free cloning using oligonucleotides CGCCACTGCAGGGATTATAAAGATGACGATGACAAGGCTAACTCCGCCCAGAAACCGGCG and CGAGGTCGACGGTATCGATAAGCTTGATATCGAATTCTAGTTACGCTTTTGCCTTCGGCATCTGCGAAACCAGTG (for T18) and GGCGGGCTGCAGATTATAAAGATGACGATGACAAGGCTAACTCCGCCCAGAAACCGGCG and CGAGGTCGACGGTATCGATAAGCTTGATATCGAATTCTAGTTACGCTTTTGCCTTCGGCATCTGCGAAACCAGTG (for T25). Substitutions have been introduced by quick change site-directed mutagenesis using complementary pairs of oligonucleotides (listed in Table S1). All constructs have been verified by restriction analyses and DNA sequencing (Eurofins, MWG).

### Bacterial Two-Hybrid Assay

The adenylate cyclase-based bacterial two- hybrid technique [58] was used as previously published [63]. Briefly, pairs of proteins to be tested were fused to the isolated T18 and T25 catalytic domains of the *Bordetella* adenylate cyclase. After transformation of the two plasmids producing the fusion proteins into the reporter BTH101 strain, plates were incubated at 30°C for 48 hours. Three independent colonies for each transformation were inoculated into 600 µl of LB medium supplemented with ampicillin, kanamycin and IPTG (0.5 mM). After overnight growth at 30°C. 10 µl of each culture were dropped onto MacConkey and LB supplemented with Bromo-Chloro-Indolyl-Galactopyrannoside (X-Gal) plates and incubated for 16 hours at 30 °C. The experiments were done at least in triplicate and a representative result on LB-X-Gal plate is shown.

### Hcp release assay

The presence of Hcp in culture supernatants has been tested as previously described [25]. Briefly, 2×10^9^ cells producing FLAG epitope-tagged Hcp were harvested and collected by centrifugation at 2,000×*g* for 5 min. The supernatant fraction was then subjected to a second low-speed centrifugation and then at 16,000×*g* for 15 min. The supernatant was then filtered on sterile polyester membranes with a pore size of 0.2 µm (membrex 25 PET, membraPure GmbH) before precipitation with trichloroacetic acid (TCA, 15% final concentration). Cells and precipitated supernatants were then resuspended in loading buffer and analyzed by SDS-PAGE and immunoblotting with the anti-FLAG antibody. As control for lysis, Western blot were immunostained with antibodies raised against the periplasmic TolB protein.

### Fluorescence microscopy and image treatment

Fluorescence microscopy experiments to follow T6SS sheath assembly have been performed as previously described [13], [33]. Briefly, bacterial cells carrying plasmid pBAD33-TssB1–sfGFP (encoding the TssB1 protein fused to a C-terminal superfolder Green Fluorescent Protein, [33]) or derivatives were grown overnight in LB medium and then diluted to an OD_600 nm_ ∼0.05 into M9 minimal medium supplemented with glycerol, casamino acids, chloramphenicol and 10% of LB. When the OD_600 nm_ reached ∼1.0, cells were harvested and washed in phosphate buffered saline (PBS), resuspended in PBS to an OD_600 nm_ ∼50, and spotted on a thin pad of 1.5% agarose in PBS, covered with a cover slip and incubated for one hour at 37°C prior to microscopy acquisition. For each experiment, ten independent fields were manually defined with a motorized stage (Prior Scientific) and stored (X, Y, Z, PFS-offset) in a custom automation system designed for time-lapse experiments. Fluorescence and phase contrast micrographs were captured every 30 sec. using an automated and inverted epifluorescence TE2000-E-PFS (Nikon, France) microscope equipped with Perfect Focus System (PFS). PFS automatically maintains focus so that the point of interest within a specimen is always kept in sharp focus at all times despite mechanical or thermal perturbations. Images were recorded with a CoolSNAP HQ 2 (Roper Scientific, Roper Scientific SARL, France) and a 100×/1.4 DLL objective. Excitation light was emitted by a 120 W metal halide light. The sfGFP images were recorded by using the ET-GFP filter set (Chroma 49002) using an exposure time of 100–200 ms. Phase contrast and fluorescence were adjusted and merged using ImageJ 1.46j (http://rsb.info.nih.gov/ij/). Fluorescence levels have been measured using a TECAN microplate reader as previously published [11], [64].

### Miscellaneous

Proteins resuspended in loading buffer were subjected to sodium dodecyl sulphate (SDS)-polyacrylamide gel electrophoresis (PAGE). For detection by immunostaining, proteins were transferred onto nitrocellulose membranes, and immunoblots were probed with primary antibodies, and goat secondary antibodies coupled to alkaline phosphatase, and developed in alkaline buffer in presence of 5-bromo-4-chloro-3-indolylphosphate (BCIP) and nitroblue tetrazolium (NBT). The anti-TolB polyclonal antibodies are from our laboratory collection while the anti-FLAG monoclonal antibody (clone M2, Sigma-Aldrich) and alkaline phosphatase-conjugated goat anti-rabbit and anti-mouse antibodies (Millipore) have been purchased as indicated.

## Supporting Information

Table S1 **Oligonucleotides used for site-directed mutagenesis.**
(DOCX)
