# Supplementary material for: Dissection of the TssB-TssC Interface during Type VI Secretion Sheath Complex Formation
Source: PLoS One. 2013 Nov 25;8(11):e81074. doi: 10.1371/journal.pone.0081074 (PMC3840085; doi:10.1371/journal.pone.0081074)
Supplement: Table S1 — Oligonucleotides used for site-directed mutagenesis. (DOCX) [file pone.0081074.s001.docx]

**Dissection of the TssB-TssC interface during Type VI secretion sheath complex formation.**

X. Y. Zhang, Y. R. Brunet, L. Logger, B. Douzi, C. Cambillau, L. Journet, and E. Cascales

**Table S1**

**Oligonucleotides used for site-directed mutagenesis of EAEC TssB1**

Helix deletion (amino-acids E104 to L130) ^a^

CCGCAATATGAACGATTTCACGCCA*GAT*AACGTGACTTTCCGTAAAGAG

CTCTTTACGGAAAGTCACGTT*ATC*TGGCGTGAAATCGTTCATATTGCGG

Amino-acid substitutions (to tryptophan) ^b^

Val-106

CGATTTCACGCCAGAGCAGTGGGCCCGACAGATTCCCCG

CGGGGAATCTGTCGGGCCCACTGCTCTGGCGTGAAATCG

Ile-110

GAGCAGGTCGCCCGACAGTGGCCCCGTCTTAAAGCCATG

CATGGCTTTAAGACGGGGCCACTGTCGGGCGACCTGCTC

Leu-117

CCCGTCTTAAAGCCATGTGGGCCATGCGTAGCCTTC

GAAGGCTACGCATGGCCCACATGGCTTTAAGACGGG

Val-106 & Ile-110

CACGCCAGAGCAGTGGGCCCGACAGTGGCCCCGTCTTAAAGCC

GGCTTTAAGACGGGGCCACTGTCGGGCCCACTGCTCTGGCGTG

Arg-108

GCCAGAGCAGGTCGCCTGGCAGATTCCCCGTCTTAAAG

CTTTAAGACGGGGAATCTGCCAGGCGACCTGCTCTGGC

Arg-112

CGCCCGACAGATTCCCTGGCTTAAAGCCATGCTGGCC

GGCCAGCATGGCTTTAAGCCAGGGAATCTGTCGGGCG

Arg-108 & Arg-112

GAGCAGGTCGCCTGGCAGATTCCCTGGCTTAAAGCCATGCTG

CAGCATGGCTTTAAGCCAGGGAATCTGCCAGGCGACCTGCTC

Leu-123

CTGGCCATGCGTAGCCTTTGGCGGGACCTGAAAGCC

GGCTTTCAGGTCCCGCCAAAGGCTACGCATGGCCAG

Leu-130

GGACCTGAAAGCCAATCTGTGGGATAACGTGACTTTCCG

CGGAAAGTCACGTTATCCCACAGATTGGCTTTCAGGTCC

a Codon upstream the deletion underlined, codon downstream the deletion italicized.

b Mutagenesized codon underlined.
